# Medical Students’ Acceptance of Tailored e–Mental Health Apps to Foster Their Mental Health: Cross-Sectional Study

**DOI:** 10.2196/58183

**Published:** 2025-01-24

**Authors:** Catharina Grüneberg, Alexander Bäuerle, Sophia Karunakaran, Dogus Darici, Nora Dörrie, Martin Teufel, Sven Benson, Anita Robitzsch

**Affiliations:** 1 Clinic for Psychosomatic Medicine and Psychotherapy, LVR-University Hospital, University of Duisburg-Essen, Virchowstraße 174, Essen, 45147, Germany, 49 201438755212; 2 Center for Translational Neuro- and Behavioral Sciences, University of Duisburg-Essen, Essen, Germany; 3 Institute of Anatomy and Neurobiology, University of Münster, Münster, Germany; 4 Institute for Medical Education, University Hospital Essen, University of Duisburg-Essen, Essen, Germany

**Keywords:** eHealth, medical education, medical students, tailored interventions, UTAUT, intention to use, e–mental health apps, app, foster, cross-sectional study, mental health problems, physician, well-being, mobile apps, acceptance, assessment, mental health apps

## Abstract

**Background:**

Despite the high prevalence of mental health problems among medical students and physicians, help-seeking remains low. Digital mental health approaches offer beneficial opportunities to increase well-being, for example, via mobile apps.

**Objective:**

This study aimed to assess the acceptance, and its underlying predictors, of tailored e–mental health apps among medical students by focusing on stress management and the promotion of personal skills.

**Methods:**

From November 2022 to July 2023, a cross-sectional study was conducted with 245 medical students at the University of Duisburg-Essen, Germany. Sociodemographic, mental health, and eHealth-related data were assessed. The Unified Theory of Acceptance and Use of Technology (UTAUT) was applied. Differences in acceptance were examined and a multiple hierarchical regression analysis was conducted.

**Results:**

The general acceptance of tailored e–mental health apps among medical students was high (mean 3.72, SD 0.92). Students with a job besides medical school reported higher acceptance (*t*_107.3_=–2.16; *P*=.03; *P*_adj_=.027; Cohen *d*=4.13) as well as students with higher loads of anxiety symptoms (*t*_92.4_=2.36; *P*=.02; *P*_adj_=.03; Cohen *d*=0.35). The *t* values were estimated using a 2-tailed *t* test. Regression analysis revealed that acceptance was significantly predicted by anxiety symptoms (β=.11; *P*=.045), depressive symptoms (β=–.11; *P*=.05), internet anxiety (β=–.12; *P*=.01), digital overload (β=.1; *P*=.03), and the 3 UTAUT core predictors—performance expectancy (β=.24; *P*<.001), effort expectancy (β=.26; *P*<.001), and social influence (β=.43; *P*<.001).

**Conclusions:**

The high acceptance of e–mental health apps among medical students and its predictors lay a valuable basis for the development and implementation of tailored e–mental health apps within medical education to foster their mental health. More research using validated measures is needed to replicate our findings and to further investigate medical students’ specific needs and demands regarding the framework of tailored e–mental health apps.

## Introduction

### Background

Medical students have a heightened incidence of mental health problems, namely anxiety [1,2] and depression [3-5], and are confronted with stressful situations throughout their careers [6,7]. Elevated levels of depression and anxiety among medical students and physicians exert considerable influence on personal well-being and patient safety [8], emphasizing the urgent need for targeted preventive and support programs [7,9-11]. The necessity for assessable and easily accessible interventions to foster mental health and well-being is of utmost importance in the medical student population [12,13].

In recent years, the surge in the significance of digitalization within health care and medical education has been noteworthy [14-16]. This trend has been particularly pronounced during the COVID-19 pandemic and persists afterward [17]. A report disseminated by a German health insurance entity in 2023 scrutinized students’ health, with a specific focus on postpandemic developments and the pivotal role of digital education and instruction [18]. The report underscored the critical importance of stress prevention and mental health initiatives [18]. Digital mental health approaches present promising avenues for surmounting barriers and enhancing the use of mental health support, for example, through mobile apps [13,19,20].

Analyzing factors influencing the acceptance of a mobile app is essential, and further research on actual uptake, adoption, and adherence is needed [21-26]. Incorporating future users directly into the development process is crucial for optimizing the adherence of new technologies and should be focused within research [27,28].

Few studies have delved into e–mental health promotion and the prevention of psychological distress among medical students and have shown that uptake of mental health support remains low due to barriers such as mental health stigma or data safety [6,29-31]. To date and to the best of our knowledge, no study has examined the acceptance of tailored e–mental health apps among medical students using a validated model. For this reason, the Unified Theory of Acceptance and Use of Technology (UTAUT) was applied in this study to lay the foundation for the development of an application especially tailored to the students’ needs and demands to foster mental health by focusing on stress management and promotion of personal skills at University Duisburg-Essen. The UTAUT evaluates the acceptance of technological systems consisting of 4 primary predictors—performance expectancy (PE), effort expectancy (EE), social influence (SI), and facilitating conditions (FC)—and has been adjusted to investigate the acceptance of eHealth interventions along with their underlying factors [23-25]. Numerous studies have used the UTAUT framework in the context of eHealth interventions among different samples [32-35].

### Objectives

Due to the evident progression of digitalization and its concomitant potential to enhance mental health while simultaneously acknowledging the existing impediments to leveraging these opportunities, this study is specifically oriented toward investigating the acceptance of tailored e–mental health apps and their foundational predictors among medical students, using the validated UTAUT model as the analytical framework.

While prior research has underscored the significance of promoting mental health among medical students [7,9,12,31], limited attention has been given to evaluate e–mental health approaches focusing on the promotion and the prevention of psychological distress among medical students using validated measures, such as the UTAUT model, and tailored approaches [28,36,37].

This study will address the following research questions: (1) What is the extent of acceptance of e–mental health apps among medical students? (2) Are there differences in acceptance among medical students based on sociodemographic and mental health data? (3) What factors predict acceptance among medical students?

## Methods

### Study Design and Participants

A cross-sectional study was conducted to assess acceptance and to analyze drivers and barriers of tailored e–mental health apps among medical students. The study was presented to medical students in the 5th year at the Medical Faculty of University Duisburg-Essen, North-Rhine-Westphalia, Germany, during the course of psychosomatic medicine. Following the course, students were given the opportunity to participate voluntarily. The participants of the study were recruited from November 2022 to July 2023. Of the 305 students attending the course, 245 (80.3%) students gave their informed consent to participate in the study. Of the 245 participants, 16 (6.5%) participants were eliminated from the sample because of missing data. In total, 229 (93.5%) students were included in the final data analysis. We applied no inclusion or exclusion criteria. Medical students were invited to participate in the study through direct contact in the context of psychosomatic medicine courses. All participants were aged 18 years or above.

### Ethical Considerations

The study was conducted in accordance with the Declaration of Helsinki and has been approved by the ethics committee of the Medical Faculty of the University of Duisburg-Essen (21‐10196-BO). Participation was anonymous, voluntary, and without any compensation. Prior to the start of the questionnaire, written informed consent was obtained and the students received background information on the purpose of the study.

### Assessment Instruments

The survey consisted of a paper-pencil questionnaire with self-developed items. Additionally, validated scales were used. The measures encompassed sociodemographic, eHealth-related, and mental health data. The primary outcome was the acceptance of an e–mental health app by using the conceptual framework of the UTAUT model’s theory.

### Sociodemographic Data

Sociodemographic data contained age, gender, marital status, employment besides medical school (occupational status), and working hours per week (0‐5, 5‐10, 10‐15, and >15 hours).

### Mental Health Data

To obtain mental health data, the validated PHQ-4 (Patient Health Questionnaire-4) measure consisting of two 2-item measures—PHQ-2 (symptoms of depression, Patient Health Questionnaire-2) and GAD-2 (symptoms of general anxiety disorder, Generalized Anxiety Disorder-2)—were used [38,39]. Answers were given on a 4-point Likert scale (0=“never” to 3=“nearly every day”). A cutoff score of 3 or more is described to be an indicator of depression (PHQ-2) [38] or general anxiety (GAD-2) [40]. Internal consistencies measured by the Cronbach α were sufficient with α=0.82 (95% CI 0.76‐0.87) for GAD-2 and α=0.81 (95% CI 0.73‐0.86) for PHQ-2. Self-generated questions were used to assess life quality (0=“very low” to 10=“very good”), mental health (0=“very low” to 10=“very good”), physical health (0=“very low” to 10=“very good”), and importance of promoting mental well-being (0=“not important” to 10=“very important”) on numerical rating scales.

### eHealth-Related Data

eHealth-related data were assessed by measuring digital overload, internet anxiety, and digital competence. Internet anxiety and digital overload were both measured on a 5-point Likert scale (1=“strongly disagree” to 5=“strongly agree”). Internal consistency measured by the Cronbach α was low to sufficient with α=0.68 (95% CI 0.6‐0.75) for the digital overload scale and sufficient with α=0.81 (95% CI 0.72‐0.87) for the internet anxiety scale. These scales were previously published and established [34,35,41,42]. Digital competence was measured with a numerical rating scale (0=“low” to 10=“high”).

### Acceptance and UTAUT Predictors

To assess medical students’ acceptance of using tailored e–mental health apps, a modified UTAUT questionnaire [24] was applied. The adapted UTAUT model consisted of 14 items and measured items on a 5-point Likert scale (1=“strongly disagree” to 5=“strongly agree”). Acceptance, operationalized as behavioral intention (BI) to use technology, is forecasted by PE, EE, and SI [25]. PE reflects the individual’s belief in the benefits they will derive from using the technology. EE signifies the perceived ease of use. SI gauges the extent to which an individual believes that their relatives or friends would endorse the use of the technology. Four items were used to assess BI and PE. Acceptance, operationalized as BI, represented the dependent variable. Two predictors of acceptance—EE and SI—were measured with 3 items each. Internal consistency (Cronbach α) was excellent for BI (α=0.91, 95% CI 0.89‐0.93) and PE (α=0.92, 95% CI 0.89‐0.94), sufficient for SI (α=0.83, 95% CI 0.77‐0.87), and low to sufficient for EE (α=.67, 95% CI 0.57‐0.75).

### Statistical Analysis

For data and statistical analysis, SPSS Statistics version 26 (IBM Corp) and R through RStudio version 4.3.1 (The R Foundation for Statistical Computing; Posit Software) were used. The raw data were collected from the survey, extracted, and processed. Relevant assumptions and prerequisites were tested prior to any statistical test [43-46]. The level of significance was set at α=0.05 for all tests. To minimize α error inflation for multiple comparisons Bonferroni correction was used and *P* values were adjusted. Sum scores (PHQ-4 scale, PHQ-2 scale, and GAD-2 scale) and mean scores (internet anxiety and digital overload) were computed. Mean scores for the UTAUT model were computed: BI, PE, EE, and SI. Consistent with previous research, acceptance scores, operationalized as BI, were categorized as “low acceptance” from 1 to 2.34, “moderate acceptance” from 2.35 to 3.67, and “high acceptance” from 3.68 to 5 [33,41,47]. Descriptive statistics (percentage and absolute count, mean scores, distributions, and standard deviations) of scales, items, and acceptance categories were performed. Additionally, explorative data analysis was conducted. Internal consistencies such as the Cronbach α and item-total correlation were calculated for scales. The normal distribution of the dependent variable (acceptance) was tested graphically and by the Kolmogorov-Smirnov test. Although violations against normal distribution were detected, parametric tests could be used according to the central limit theorem (n>30) and the robustness of the *t* test and Welch-ANOVA against normal distribution violations [44]. Means of acceptance were compared between groups using the *t* test (occupational status, PHQ-2, and GAD-2) and Welch-ANOVA (gender and marital status). The predictive model of acceptance was tested using multiple hierarchical regression analyses. The following predictors were included stepwise: sociodemographic data, mental health data (PHQ-2 and GAD-2), eHealth-related data, and the UTAUT core predictors (EE, SI, and PE). Linearity could be assumed and was analyzed using a scatter plot of the residuals against fitted values. Multicollinearity was not detected because all values of the variance inflation factor were <5. The normality of residuals could be assumed due to the central limit theorem. Homoscedasticity was proven and analyzed using a scatter plot of the standardized residuals and adjusted predicted values. According to Cohen *d*, effect sizes were reported and interpreted as small (0.2), medium (0.5), and large (0.8) [48].

## Results

### Study Population

In this sample, participants’ age ranged from 20 to 37 years (mean 25.05, SD 2.82 years). Medical students experienced low digital overload (mean 2.85, SD 0.92) and low internet anxiety (mean 1.72, SD 0.79). Digital competence was high among medical students (mean 6.97, SD 1.72; range 0‐10). For detailed characteristics, see Table 1.

**Table 1.** Sociodemographic and mental health data of participants (n=229).

Variable		N (%)	Mean (SD)	Acceptance, n (%)		
				Low^a^	Moderate^b^	High^c^
**Gender**						
	Woman	157 (68.6)	—^d^	13 (8.3)	42 (26.8)	102 (65)
	Man	70 (30.6)	—	9 (12.9)	21 (30)	40 (57.1)
	Nonbinary	2 (0.9)	—	0 (0)	2 (100)	0 (0)
**Marital status (n=228)**						
	Single, divorced, or separated	139 (61)	—	14 (10.1)	45 (32.4)	80 (57.6)
	Married or in a relationship	89 (39)	—	8 (9)	20 (22.5)	61 (68.5)
**Job**						
	Yes	165 (72.1)	—	14 (8.5)	43 (26.1)	108 (65.5)
	No	64 (28)	—	8 (12.5)	22 (34.4)	34 (53.1)
**Working hours per week (n=166)**						
	0‐5	37 (22.3)	—	2 (5.4)	9 (24.3)	26 (70.3)
	5‐10	84 (50.6)	—	8 (3.8)	21 (25)	55 (65.5)
	10‐15	25 (15.1)	—	2 (8)	7 (28)	16 (64)
	>15	20 (12.1)	—	2 (10)	7 (35)	11 (55)
Mental health^e^		—	6.82 (1.72)	7.4 (2.2)	6.9 (2.3)	6.7 (2.4)
Physical health^e^		—	8.00 (1.84)	8.5 (1.6)	8 (1.8)	7.9 (1.9)
Life quality^e^		—	7.92 (1.67)	8.4 (1.1)	7.9 (1.7)	7.9 (1.7)
Promotion of mental well-being^e^		—	8.68 (1.80)	7.9 (2.3)	7.9 (1.7)	9 (1.7)
**PHQ-2^f^ score (range 0‐6)**		—	1.26 (1.42)	—	—	—
	Low (≤2)	201 (87.8)	0.84 (0.84)	21 (10.5)	57 (28.4)	123 (61.2)
	High (≥3)	28 (12.2)	4.25 (1.11)	1 (1.7)	8 (28.6)	19 (67.9)
**GAD-2^g^ score (range 0‐6)**		—	1.85 (1.51)	—	—	—
	Low (≤2)	178 (77.7)	1.19 (0.76)	20 (11.2)	52 (29.2)	106 (59.6)
	High (≥3)	51 (22.3)	4.16 (1.17)	2 (3.9)	13 (25.5)	36 (70.6)

a Low acceptance, with scores ranging from 1 to 2.34.

b Moderate acceptance, with scores ranging from 2.35 to 3.67.

c High acceptance, with scores ranging from 3.68 to 5.

d Not applicable.

e Higher scores indicate higher levels of mental health, physical health, life quality, or importance of promoting mental well-being (range 0‐10).

f PHQ-2: Patient Health Questionnaire-2.

g GAD-2: Generalized Anxiety Disorder-2.

### Acceptance of Tailored e–Mental Health Apps

The general acceptance of tailored e–mental health apps among medical students was high (mean 3.72, SD 0.92). Dividing the acceptance categories from low to high, 62% (142/229) participants showed high acceptance (mean 4.31, SD 0.45), 28.4% (65/229) showed moderate acceptance (mean 3.11, SD 0.28), and 9.6% (22/229) showed low acceptance (mean 1.76, SD 0.42).

Between groups, significant differences in acceptance were identified between occupational status (*t*_107.3_=–2.16; *P*=.03; *P*_adj_=.03; Cohen *d*=4.13) and GAD-2 groups (*t*_92.4_=2.36; *P*=.02; *P*_adj_=.03; Cohen *d*=0.35) using a 2-tailed *t* test. Students with a job besides medical school reported higher acceptance of tailored e–mental health apps than students without a job. Medical students with high GAD-2 levels (high load of anxiety symptoms) showed higher acceptance than students with low GAD-2 levels (low load of anxiety symptoms). No significant differences between acceptance were found regarding PHQ-2 groups (low and high), gender (female, male, and divers), and marital status via ANOVA and *t* test (*P*_adj_>.5).

### Hierarchical Linear Regression Analysis and Predictors of Acceptance

A hierarchical linear regression analysis was conducted to evaluate predictors of acceptance among medical students regarding tailored e–mental health apps.

Sociodemographic data were included in the first step, explaining 3.6% of the variance in acceptance (*R*^2^=0.036; *R*^2^_adj_=0.022; *F*_3,222_=2.72; *P*=.045). Occupational status emerged as a significant positive predictor (β=.31; *P*=.03).

In the second step, mental health data were added to the analysis, increasing the explained variance to 6.4% (*R*^2^=0.064; *R*^2^_adj_=0.042; *F*_5,220_=2.99; *P*=.01). GAD-2 was identified as a significant predictor (β=.12; *P*=.03) of acceptance.

In the third step, eHealth-related data were added to the model, which further explained 8.2% of the variance in acceptance (*R*^2^=0.082; *R*^2^_adj_=0.048; *F*_8,217_=2.14; *P*=.02).

In the fourth and final step, the UTAUT predictors (EE, PE, and SI) were added (overall model), resulting in a comprehensive model that explained 65.8% of the variance in acceptance (*R*^2^=0.658; *R*^2^_adj_=0.647; *F*_11,214_=37.47; *P*<.001). The following variables (UTAUT core predictors) showed a significant positive prediction: UTAUT PE (β=.22, *P*<.001), UTAUT EE (β=.32, *P*<.001), and UTAUT SI (β=.44; *P*<.001).

To sum up, within the overall model, the UTAUT predictors, PHQ-2 and GAD-2 sum scores, internet anxiety, and digital overload were associated with the acceptance of tailored e–mental health apps among medical students. For a detailed overview of the hierarchical regression model of acceptance, see Table 2.

**Table 2.** Hierarchical regression model of acceptance (the extended Unified Theory of Acceptance and Use of Technology model; n=226).

Predictors		β^a^	β^b^	*t* ^c^	*R* ^2^ ^d^	∆*R*^2^^e^	*P* value
Intercept		–.22	–.00	–0.46	—^f^	—	.64
**Step 1: Sociodemographic data**		—	—	—	0.036	0.036	—
	Gender	.04	.02	0.53	—	—	.59
	Age	.01	.03	0.80	—	—	.43
	Occupational status	.16	.08	1.78	—	—	.08
**Step 2^g^: Mental health data**		—	—	—	0.064	0.028	—
	PHQ-2^h^, sum score	–.07	–.11	–1.98	—	—	.05
	GAD-2^i^, sum score	.07	.11	2.02	—	—	.04
**Step 3^g^: eHealth-related data**		—	—	—	0.082	0.018	—
	Digital overload	.10	.10	2.18	—	—	.03
	Internet anxiety	–.14	–.12	–2.49	—	—	.01
	Digital competence	.01	.03	0.47	—	—	.64
**Step 4^g^: UTAUT^j^ core predictors**		—	—	—	0.658	0.576	—
	Social influence	.44	.43	7.57	—	—	<.001
	Performance expectancy	.22	.24	4.31	—	—	<.001
	Effort expectancy	.32	.26	5.24	—	—	<.001

a Unstandardized coefficient beta.

b Standardized coefficient beta.

c Test statistics were estimated using a 2-tailed *t* test.

d Multiple *R*2 reported, determination coefficient.

e Changes in *R*2.

f Not applicable.

g In steps 2, 3, and 4, only the newly included variables are presented.

h PHQ-2: Patient Health Questionnaire-2.

i GAD-2: Generalized Anxiety Disorder-2.

j UTAUT: Unified Theory of Acceptance and Use of Technology.

## Discussion

### Principal Findings

This study focused on examining the acceptance of tailored e–mental health apps and the factors influencing their use to promote medical students' mental health.

The general acceptance was high. Students with a job besides medical school reported higher acceptance as well as students with higher loads of anxiety symptoms. Acceptance was significantly predicted by occupational status, anxiety symptoms, depressive symptoms, internet anxiety, digital overload, and the 3 UTAUT core predictors—PE, EE, and SI.

The participants in this sample reported higher overall acceptance compared to previous research involving different target groups [23,33,42]. A qualitative study conducted by Dederichs et al [12] corroborates our findings, elucidating universally positive perspectives among medical students regarding internet- and mobile-based interventions. Preceding investigations have posited that augmented levels of educational attainment are concomitant with elevated acceptance scores [32,49], concurrently accentuating the advantages of e–mental health methodologies, including their low-threshold nature, temporal flexibility, and provision of anonymous support [12].

Among our cohort, self-rated promotion of mental well-being was highly valued, indicating general interest in mental health promotion as an important prerequisite and determinant of increasing acceptance.

The UTAUT core predictors elucidated the majority of the variance in acceptance, substantiating the model’s efficacy in appraising e–mental health acceptance among medical students and aligning with antecedent research [25,33,42]. Despite prior investigations indicating age [25,32,42] and gender [25,49] as salient determinants influencing acceptance within heterogeneous populations, these variables did not achieve statistical significance in this study. This lack of significance may be attributed to the existence of comparable stress factors affecting all participants uniformly.

A notable proportion, 12.2% (28/229), displayed indicators suggestive of depressive symptoms (PHQ-2), while 22.3% (51/229) exhibited symptoms indicative of a general anxiety disorder (GAD-2). These findings are consistent with extant research documenting the psychological vulnerability of medical students, illustrating elevated levels of anxiety and depression [1,6,50]. This underscores the imperative for psychological support interventions [2,3,10]. Our analysis revealed that mental health data concerning anxiety symptoms positively predicted acceptance within our model, aligning with prior research [51]. In contrast to that, depressive symptoms were associated with lower acceptance within our model. The acceptability may be decreased among students with higher depressive symptoms due to fear of additional loads. Furthermore, barriers, such as mental health stigma or data safety, were described as known challenges within previous research focusing on help-seeking behavior [30,36]. Additional information and educative programs or interventions may have beneficial effects to increase help-seeking and decrease stigma [29,52-55], but their impact needs to be investigated further.

Students concurrently managing part-time employment and medical school responsibilities demonstrated higher acceptance scores. Research specifically focusing on the mental health of working medical students is scarce [9,56]. Based on the findings, we would suggest that the additional load due to a part-time job results in higher acceptance levels of mental health support programs but this needs to be investigated further.

A study by Joiner et al [57] found that individuals born after 1993 exhibited lower internet anxiety and higher internet identification, reinforcing our findings. In our sample, most of the participants were born in the 1990s and 2000s. Internet anxiety and digital overload were observed at low levels and significant predictors of acceptance in the overall regression model. Aligning with previous research [23], high levels of internet anxiety were associated with decreased acceptance.

Digital competence was high within our sample. High internet identification and regular use of digital media might have influenced digital competence within our sample. Information on digital skills [58], preventive strategies, and digitalization need to be integrated further within medical education [15].

While acceptance and potential usage constitute crucial prerequisites for the implementation of digital approaches [23,59], it is imperative to acknowledge additional factors, including barriers and risks associated with the promotion of such approaches. Notably, skepticism and a lack of knowledge regarding e–mental health apps among medical students underscore the necessity for augmented information dissemination and increased personal experience with digital health approaches [22,36]. Attention must be directed toward addressing stigma and concerns related to data security [30,36]. Comprehensive assessments of additional barriers influencing actual usage and dropout rates are warranted in the implementation of e–mental health approaches [19,60].

The outcomes of this study establish a foundational framework for subsequent research endeavors and the implementation of e–mental health apps within the realm of medical education. The imperative for further implementation and rigorous evaluation of digital interventions for medical students is underscored.

### Limitations

This study has limitations that should be considered when interpreting the presented results. It should be noted that studies assessing medical students’ acceptance with validated instruments are still scarce and comparability is limited. The cross-sectional design does not allow causal inferences. Overall, overrepresentation may diminish representativeness, generalizability, and external validity, which is a common bias in research. In the context of a tailored design approach, additional stakeholders should be integrated into future studies [61]. The intention-behavior gap should be considered, as our study assessed theoretical willingness rather than actual usage. Within this study, the Cronbach α, a conservative measure assessing reliability, was used, and it should be noted that the Cronbach α of the EE scale and digital overload scale were lower compared to those observed in previous studies [33,35,41,42]. One possible explanation may be inconsistent response patterns; therefore, the interpretation should be done with caution. According to previous studies [21-28], adherence, actual usage, and dropout rates of e–mental health approaches should be investigated further. While the 3 fundamental predictors of the UTAUT model—EE, PE, and SI—remain crucial, additional factors should be focused on to comprehensively grasp and optimize acceptance levels further.

### Conclusions

In this investigation, the focus was on evaluating the acceptance of tailored e–mental health apps and its influencing factors in promoting medical students’ mental health. The overall acceptance was found to be high, with students having part-time jobs alongside medical school and students with elevated anxiety levels reporting even higher levels of acceptance. Besides the 3 UTAUT core predictors (PE, EE, and SI), additional significant predictors influence acceptance among medical students including occupational status, anxiety symptoms, depressive symptoms, internet anxiety, and digital overload. As digitalization transforms the medical sector, integrating supportive digital tools into medical education requires a focus on promoting a healthy learning environment and well-being among future physicians. Preventive strategies, including addressing barriers like stigma, are crucial. This study contributes valuable insights in order to develop and implement a digital application to foster medical students’ mental health focusing on stress management and promotion of personal skills at Medical University Duisburg-Essen, Germany.

## Abbreviations

BI: behavioral intention
EE: effort expectancy
FC: facilitating conditions
GAD-2: Generalized Anxiety Disorder-2
PE: performance expectancy
PHQ-2: Patient Health Questionnaire-2
PHQ-4: Patient Health Questionnaire-4
SI: social influence
UTAUT: Unified Theory of Acceptance and Use of Technology
